# Opposing impacts of DNA polyplex crosslinking on delivery efficiency and vaccine responses

**DOI:** 10.1016/j.omtn.2025.102656

**Published:** 2025-08-08

**Authors:** Satoshi Uchida

**Affiliations:** 1 Department of Advanced Nanomedical Engineering, Medical Research Laboratory, Institute of Integrated Research, Institute of Science Tokyo, 1-5-45 Yushima, Bunkyo-ku, Tokyo 113-8510, Japan; 2 Innovation Center of NanoMedicine (iCONM), Kawasaki Institute of Industrial Promotion, 3-25-14 Tonomachi, Kawasaki-ku, Kawasaki 210-0821, Japan

## Main text

In a recent issue of *Molecular Therapy Nucleic Acids*, Sallah et al. developed polymeric nanoparticle-based plasmid DNA (pDNA) vaccines targeting influenza viruses.^1^ During their research, they discovered that inducing sufficient inflammatory responses—rather than merely achieving efficient antigen protein expression—may be critical for enhancing vaccine efficacy. Several reports on mRNA vaccines have shown a limited correlation between antigen expression efficiency and vaccination outcomes.^2^^,^^3^^,^^4^ However, the underlying mechanisms remain poorly understood. Through detailed mechanistic analyses of factors affecting pDNA vaccines, this study offers valuable insights into the design rationale of nucleic acid-based vaccines. Furthermore, the researchers identified a formulation that achieved high protein expression efficiency but low antibody induction, which could be useful for therapeutic protein production beyond vaccination purposes.

During the COVID-19 pandemic, mRNA vaccines were rapidly developed, demonstrating high safety, efficacy, and scalability for billions of doses. However, their requirement for ultra-low temperature storage and relatively high manufacturing costs have limited their distribution, especially in low-income countries. pDNA offers a promising alternative to address these challenges. Additional concerns with currently approved mRNA vaccines include the use of lipid nanoparticles (LNPs), whose pro-inflammatory nature may cause adverse effects.^4^ Moreover, LNPs have shown undesired liver accumulation even after local intramuscular injection. To address these issues, polyplexes—complexes prepared from polycations and nucleic acids—present a potential alternative. Despite their promise in nucleic acid delivery, polyplexes have been less explored for vaccine applications compared to LNPs. This study focuses on optimizing pDNA polyplexes for vaccine use.

**Figure 1:**
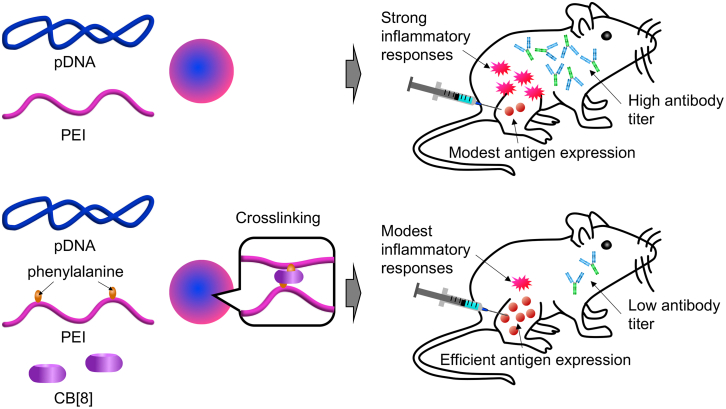
Preparation of crosslinked pDNA polyplexes and their functions in mice Upper: uncrosslinked polyplexes triggered strong inflammatory responses, leading to robust antibody production after vaccination, despite lower transfection efficiency in mice. Lower: polyplexes crosslinked via CB[8], which binds two phenylalanine moieties on PEI, exhibited moderate inflammatory responses and modest antibody production, even with higher transfection efficiency.

As a platform polycation, the study employed polyethyleneimine (PEI), a well-studied polymer known for its ability to facilitate polyplex escape from endosomes into the cytosol. While increasing the molecular weight of PEI enhances transfection efficiency, it also increases cytotoxicity. Crosslinking PEI chains is a common strategy to resolve this trade-off, as it boosts transfection efficiency by increasing the apparent molecular weight while reducing toxicity through dissociation of the crosslinks.^5^ For crosslinking, the current study used cucurbit[8]urils (CB[8]), barrel-shaped host molecules that bind two phenylalanine moieties—introduced into polycations as guest molecules—via host-guest chemistry (Figure 1).^1^

The researchers first optimized polyplex formulations by varying PEI molecular weight and phenylalanine modification efficiency, based on transfection performance. All tested polyplexes had particle sizes of 60–80 nm and zeta potentials of 20–40 mV. Transfection efficiency increased with PEI molecular weight, while excessive phenylalanine modification reduced it. The polycation-to-pDNA ratios were then adjusted to balance transfection efficiency and cytotoxicity. When applied as a vaccine against influenza viruses, the optimized formulation loading hemagglutinin (HA) pDNA induced high antibody titers following intramuscular injection. This formulation conferred protection against viral challenge, reducing lung viral load, mitigating inflammation, and minimizing body weight loss.

Mechanistic analyses then examined the impact of CB[8]-based crosslinking. Surprisingly, crosslinking reduced vaccine efficacy in terms of anti-HA antibody levels and protection against viral challenge, despite increasing *in vivo* transfection efficiency in muscle tissue (Figure 1). Compared to the crosslinked formulation, the uncrosslinked polyplexes enhanced the secretion of inflammatory molecules in cultured human monocytes, elevated serum cytokine levels, and promoted macrophage and dendritic cell infiltration into draining lymph nodes after intramuscular injection in mice. These findings suggest that the pro-inflammatory nature of uncrosslinked polyplexes may enhance vaccine efficacy, particularly in inducing humoral immunity.

The authors previously investigated the role of inflammation in self-amplifying RNA (saRNA) vaccines by comparing polyplex-based and LNP-based formulations.^3^ Although saRNA polyplexes achieved higher protein expression than LNPs, LNPs induced stronger antibody responses, presumably due to their pro-inflammatory effects. These findings underscore the potential benefits of innate immune activation in nucleic acid vaccines. However, the relationship between inflammation and vaccine efficacy is complex. Studies have shown that blocking innate immune signaling can enhance saRNA and mRNA vaccine efficacy, indicating that inflammation may hinder vaccine performance depending on the delivery system and administration route.^6^^,^^7^ The relative kinetics of innate immune activation relative to antigen presentation may be crucial for T cell activation, contributing to the opposing roles of inflammation in vaccination.

The current study further highlights the complexity of vaccine responses.^1^ While PEI crosslinking impaired antibody induction, it enhanced CD8^+^ T cell responses. These results suggest that optimal vaccine designs for inducing humoral and cellular immunity may differ. Further research is needed to unravel the mechanisms underlying nucleic acid vaccine efficacy, including the roles of innate immune signaling and nanoparticle migration to lymph nodes.

Beyond vaccines, pDNA polyplexes have potential in other therapeutic areas, such as protein replacement therapy. In these applications, antibody responses against the expressed proteins can be detrimental. Polyplexes crosslinked with CB[8] offer favorable properties for such therapies, exhibiting efficient transfection with reduced immunogenicity. With further optimization, the polyplexes developed in this study could serve as a versatile platform for both immune-stimulating vaccines and non-immunogenic protein therapies.

## Declaration of interests

The author declares no competing interests.
